# Health literacy

**DOI:** 10.3402/jchimp.v3i2.21217

**Published:** 2013-07-05

**Authors:** Robert P. Ferguson

At a Morbidity and Mortality conference recently (M&M), I was presented with a patient who was rapidly decompensating in the intensive care unit, and a decision had to be made regarding CPR, including intubation. During the hospitalization just a few hours before this, the patient had been presented with this information and elected to undergo CPR, but not to undergo intubation. During the course of the subsequent cardiac arrest, when CPR lasted for more than 90 min, the patient received respiratory assistance, but no intubation because of his advanced directives. This issue complicated the management of the patient in the intensive care unit when the case was discussed at the M&M today. We were unable to question those who made the advanced directive arrangements with this patient due to duty hour limits. All work in the critical care unit is done by shifts, and the odds of any individual being available on a given morning was less than 50%, as was the case today. In hindsight, it seems unlikely to me that this elderly gentleman understood what was being asked of him. During his advanced directives discussion, what is the proper approach in a situation like this? How much health literacy does the patient need to be able to handle this kind of cataclysmic series of circumstances in a life-threatening situation, in a strange environment, knowing nobody there?

I routinely attend around the infamous danger date of July 1st. I have been doing it for years. I feel that my role as a program director should focus on the inpatient service during the first few days with new medicine residents. I have repeatedly noted a combination of resident fears (appropriate) and unfamiliar behavior, including scattered communication skills. Policies and procedures being what they are, mean that there are many things that can be done in any circumstances to try to improve communication. However, the issue of health literacy of the individual is paramount. Health literacy is defined as a level of intelligence and communication skills that a patient must have to make informed decisions regarding what is best for them. For many years, there have been concerns about the lack of appreciation of patient health literacy. In 2006, the Institute of Medicine raised the issue of major problems in providing equal care for all (1). It has been a topic of increasing concern, as studies have frequently demonstrated that health literacy has an impact on outcomes and can lead to significant health disparities. The Healthy People 2020 Initiate of the United States Department of Health and Human Services (2) has included it as a pressing new topic, with objectives to address it in the decade to come. Furthermore, physicians and the specialty residents in training have inconsistent skills in this area. There is a belief that because of the current pressures being what they are, that this is likely to get worse before it gets better. What are the pressures?

- The helter-skelter reality of a busy impatient medical service in 2013, includes short hospital stays, rapid turn-over of staff, both physicians and others, throughput pressures, increasing technology and invasiveness, and increasing use of consultants.
- With each new computer system iteration, less talking to inpatients and more observing on computer screens have been noted in our institution.
- The lack of primary care physicians taking care of the patient in the hospital probably is as important as any other factor. This, unfortunately, is a regularly diminishing entity due to the lack of hospital visits by most office-based physicians, as well as the expansion of hospitalists on inpatient services. In my experience as Chief of Medicine in a northeastern-community hospital, at least 50% of inpatient general medical patients are now taken care of by shift-oriented hospitalists.
- On the resident service, one major factor that goes with the reality is that in at least half of the programs that we sampled during a recent survey, (3) more than two-thirds of their medical residents spoke English as a second language. My own role at the bedside of July rounds is to serve as someone who that patient, after hearing somewhat difficult to understand directives and information, can turn to as a gray-haired, English-speaking American doctor who can translate and run interference for the patient.

Although the reasons for problematic patient health literacy and the impatient service may be obvious, the question is what can be done about it. I think that it is important to train residents on the issues related to health literacy, particularly those that may have cultural or language issues. What is the right approach? The approach that we are taking in our department is to use the bedside of our teaching program. Others have noted that the majority of teaching health literacy issues occurs primarily in a lecture hall, not an ideal location, because it is not a real environment and does not face the real patient, which makes all the difference (3). It is also not sustainable, it has to be reapplied and reinforced to be sustainable. The issues related to technical jargon use are major factors. Patients do not know what an “MI” is, but they certainly know the meaning of “heart attack”. An experienced primary care physician translates into layman's terms, while the inexperienced technically oriented house officer may be bewildered by it. Faculty development is an important component here; there is no better role model than a bedside-experienced faculty member with health literacy awareness skills. This can have a great effect. We need to do something that is sustainable and something that can be measured. What we will do in our program in the future is to reevaluate residents after they have health literacy exposure, and hopefully patients as well.

Getting back to this patient and what could have been done differently. It is a complex case with many variables. Clearly advanced directives at a more relaxed and less stressful time would have been preferable. The midst of clinical illness in a medical intensive care unit is not the best time to make these kinds of hard choices. A more established doctor–patient relationship should be sought out, although the reality is that most, if not all, inpatient providers these days, be it the attending physicians, hospitalists, or residents in training, have a shift-oriented mentality. I was not at the bedside, nor anyone else during the presentation at M&M. Nevertheless, I am confident that patient autonomy might have been more salvageable if those involved were more experienced in this type of interaction.

*Robert P. Ferguson, MD*
Editor
